# Protocol for a systematic review of economic evaluations considering costs and health outcomes of weather and climate-related extreme events in humans

**DOI:** 10.1136/bmjopen-2024-096554

**Published:** 2025-02-07

**Authors:** Saeideh Babashahi, Collins Iwuji, Kingsley Orievulu, Ekeminiabasi Eyita-Okon, Dominic Kniveton

**Affiliations:** 1 Department of Global Health and Infection, Brighton and Sussex Medical School, University of Sussex, Brighton, UK; 2 Department of Population Science, Africa Health Research Institute, Durban, South Africa; 3 Africa Health Research Institute, Durban, South Africa; 4 University of the Witwatersrand Johannesburg, Johannesburg, South Africa; 5 School of Global Studies, University of Sussex, Brighton, UK

**Keywords:** Climate Change, HEALTH ECONOMICS, Healthcare Costs, Hospitalisation, Mortality

## Abstract

**Abstract:**

**Background:**

Extreme weather and climate-related events are increasing in frequency and intensity, which pose substantial human casualties and economic losses. The healthcare and health-determining sectors require information about how extreme weather events affect the population’s health, healthcare and other sectors’ capacities to prepare for and manage these events and their aftermath. We aim to conduct a systematic review to identify the recent evidence on the costs and health outcomes of extreme weather events in humans.

**Methods and analysis:**

The Preferred Reporting Items for Systematic Reviews and Meta-Analyses Protocols guidelines were followed for reporting this protocol. A comprehensive search will be conducted using several search engines, for example, PubMed, Scopus and Institute for Scientific Information Web of Science. Peer-reviewed and grey literature published in English that evaluated the health outcomes and costs of extreme climate events will be retrieved without restriction on the publication year or geographical location. Two reviewers will independently assess each study for inclusion. Study quality will be evaluated with the recommended quality assessment tools. Data will be reported using descriptive statistics, graphical plots and a narrative synthesis.

**Ethics and dissemination:**

An ethical assessment was not required. The data generated from the systematic review will be disseminated through peer-reviewed journal articles and international conferences and will inform our original research study.

**PROSPERO registration number:**

This systematic review has been registered at the International Prospective Register of Systematic Reviews (registration ID: CRD42024582635).

**STRENGTHS AND LIMITATIONS OF THIS STUDY**

- The systematic review will be limited to any type of economic evaluation studies only.
- The review will be conducted without restriction on geographical area, context or publication date.
- We will follow the Preferred Reporting Items for Systematic Reviews and Meta-Analyses statement to improve the transparency and rigour of our methodology.
- We will search several large databases covering both peer-reviewed and grey literature.
- Our systematic review wil be limited to studies published only in English.

## Introduction

 Climate change refers to long-term seasonal changes in temperature and weather patterns, which may manifest in the form of extreme weather and climate events, for example, droughts, floods, freezes and heatwaves.^1 2^ Extreme climate events’ frequency, intensity and modification to their extent and timing have been increasing in many parts of the world.^3 4^ Risks generated from extreme weather and climate events develop from the intersection of the physical hazard (eg, rain and wind), the extent of exposure to the hazard, the vulnerability of population subgroups or communities and the capacity to prepare for and recover from extreme climate events.^4^,^6^

The impacts of extreme events are many and diverse. The changing climate has created considerable concerns in the underlying environmental, social and economic determinants of health and well-being (eg, clean air, safe drinking water, sufficient food, secure shelter, income and social protection) at the local, regional and global levels.^3 4^ Globally, the cost of damage related to climate change is projected to be between US$1.7–3.1 trillion per year by 2050, and this is expected to increase over time.^7^ This includes the cost of damage to infrastructure, properties and agriculture.^3^

Apart from the projected increase in infrastructure damage,^7^ climate extreme events can have catastrophic effects on communities and health systems and can affect the health and overall well-being of individuals.^4 8^ People can experience a wide range of physical health impacts (eg, inpatient or outpatient visits, mortality and treatment interruptions) as well as long-term impacts on mental health.^9^,^13^ The World Meteorological Organization reports that there have been nearly 12 000 disasters between 1970 and 2021, with at least two million deaths, and 90% of these disasters have occurred in developing countries due to extreme weather since the 1970s.^14^

Healthcare systems and facilities are impacted by extreme events, affecting patients, healthcare workers, medical and non-medical supplies, facility operations and critical infrastructure. To prepare for and respond to climate change, the healthcare sector and other health-determining sectors require information about how extreme weather events affect population health, healthcare delivery and the sectors’ infrastructure and the required capacities to prepare for and manage these events and their aftermath.^4 15^

Previous reviews have focused on the general adverse impacts of climate change, mainly on heat events.^16^,^19^ Other systematic reviews that have focused only on the health impacts of climate change events without considering relevant costs are limited to a geographical area or a health condition or are no longer up-to-date.^1620^,^23^ Building on a scoping review conducted by Schmitt *et al* in 2016,^16^ this is a protocol for a systematic review of the most recent evidence from a broader and more comprehensive overview of the literature (both peer-reviewed and grey literature) evaluating health-related outcomes and costs of extreme events as well as critically appraising the identified studies.

## Methods and analysis

### Eligibility criteria and research questions

We will undertake a systematic review following the Preferred Reporting Items for Systematic Reviews and Meta-Analyses (PRISMA) statement.^24^ The systematic review protocol was registered with the International Prospective Register of Systematic Reviews (registration ID: CRD42024582635). Table 1 represents the PICOS framework (participants, interventions, comparisons, outcomes and study design) used to develop the inclusion criteria and research questions, as listed below.^25^

**Table 1:** PICOS table representing inclusion and exclusion criteria

Component	Description
Population	The population of interest includes human participants (general population, individuals with underlying health conditions, their family members and/or carers) with no restriction on age or gender.
Intervention	All types of climate extreme events for which the economic and health impacts were evaluated using validated tools.
Comparator	All studies with or without comparators/controls will be eligible for inclusion in the review.
Context	All original research articles published in peer-reviewed journals and grey literature written in English with no restriction on publication date. There will be no restrictions on country or setting.
Outcome	The primary outcomes of interest will include the economic costs and health outcomes (eg, inpatient and outpatient visits, deaths, etc) related to extreme events and other evidence such as study population, context, etc. The secondary outcomes will consist of methodological and research gaps and the quality of included studies.
Study design	Any types of economic evaluation that evaluated costs and health outcomes of extreme events (peer-reviewed or grey literature) with or without a control group will be included in the analysis. All other study designs (eg, reviews, commentaries, editorials, book chapters) will be excluded.

Note: PICOS: Ppopulation, intervention, context, outcome, and study design.

The review will seek to answer the following research questions:

1. What are the methodologies and tools used for measuring the costs and health outcomes of a range of climate-related extreme events?
2. Which population groups and contexts have been assessed for the economic, health and well-being impacts of climate-related extreme events?
3. What are the main findings (eg, costs and health outcomes) reported for the extreme weather events in the included studies?
4. What are the methodological quality and reporting gaps of the identified studies and the literature?

### Search strategy

A systematic search will be conducted in the following search engines: Medline (PubMed), Scopus, Institute for Scientific Information Web of Science, Global Health (Ovid), WHO (https://iris.who.int/), OpenDOAR (grey literature), The King’s Fund (grey literature), the National Grey Literature Collection, Grey Literature Report and Google Scholar (grey literature). The search will also include a reference list screening of previous reviews and included studies (snowballing). Only original research studies published in English will be included (table 1). There will be no restrictions on the context, country, study population, gender, age, extreme weather events, health condition assessed or publication date. We will list indexing terms (eg, subject headings/subheadings) and keywords used to describe concept clusters (eg, single words or phrases that may appear in titles or abstracts in full or using truncation operators) to create a comprehensive set of likely search terms. Search terms will include (health OR clinical OR non-health OR well-being OR hospital* OR mortal* OR morbidit* OR injur*) AND (outcome* OR impact* OR consequence* OR aspect*) AND (economic* OR cost* OR loss* OR damage* OR productivit*) AND (extreme weather OR climate change OR flood* OR storm* OR extreme heat OR extreme cold OR sea rise level OR drought* OR freeze* OR cyclone*). The research team will develop the literature review search strategy with a trained and experienced librarian at Sussex and Brighton Medical School. We did a preliminary search on 26 June 2024, to ensure there is no prior systematic review on this topic. We started our literature search in January 2025 and expect to complete the systematic review by August 2025.

### Study screening and selection

All records identified from the searches will be transferred to EndNote reference manager software V.21, and all duplicates and titles in languages other than English will be removed. The review process will include an initial screening of the title and abstract of the studies to assess their eligibility for full-text retrieval using prespecified screening criteria. Any studies that were not excluded confidently through the title and abstract screening during the initial screening step will be included for full-text screening. The screening of the selected studies will be carried out independently by two reviewers. Any disagreement on selected papers will be resolved through discussion among authors. Following PRISMA guidelines, a flowchart will be created to illustrate the selection process.

### Data extraction

Data from the peer-reviewed and grey literature based on the inclusion criteria (see table 1) will be extracted by the first author in a bespoke data extraction form. The data extracted will be checked by a second reviewer for accuracy and completeness. Microsoft Excel (Microsoft 365 MSO, V.2406 build 16.0.17726.20206) will be used by the research team for charting the data from selected studies and reporting the variables regarding the study, participants, extreme climate events and their impacts—for example, authors, publication year, study context and design, sample size, extreme event type, health and their associated costs, tools used for measurement, key findings and limitations—based on the research questions. The search strategy and the study selection process, in the form of the PRISMA flowchart and data, will be publicly available and published in the systematic review.

### Quality of evidence and assessing risk of bias

Studies selected for retrieval will be independently assessed by two reviewers for methodological validity using relevant standardised appraisal instruments. As recommended by the Campbell and Cochrane Economics Methods Group,^26^ we will use the Drummond and Jefferson checklist^27^ and the Evers *et al* checklist^28^ for assessing the methodological and reporting quality of economic evaluations. Any discrepancies will be resolved through discussions within the research team.

### Data synthesis and analysis

Data synthesis will be undertaken by the first author in consultation with the broader research team. The findings of selected studies will be summarised using descriptive statistics, graphical plots and a narrative synthesis, highlighting the key research findings—for example, type of extreme event, economic and health impacts, setting, population, etc—of selected studies in the general population and individuals with underlying conditions and the existing research gaps in the literature.

In terms of other findings, we will use a narrative approach for data analysis and synthesis, due to the heterogeneity we expect to observe with respect to study designs, extreme events and health conditions assessed. If data permits, further subgroup analysis will be conducted concerning the population characteristics or heterogeneity in the study findings. This systematic review will be conducted and reported following the PRISMA guidelines.^24^

### Ethics and dissemination

An ethical assessment was not required for this protocol paper. The data generated from the systematic review will be disseminated through peer-reviewed journal articles and international conferences and will inform our original research study.

### Patient and public involvement

Patients and/or the public were not involved in the design, conduct, report or dissemination plans of this systematic review protocol.

## Discussion

This is a protocol to conduct a systematic review of the literature that aims to identify, assess and synthesise the evidence on costs and health outcomes of extreme weather events. The previous review was a scoping review of literature that included studies published up to December 2015.^16^ This systematic review aims to assess the most recent evidence on costs and health outcomes caused by extreme events from a wider body of peer-reviewed and grey literature. We will also assess the methodological quality and limitations of the included studies that have not been evaluated in the prior review by Schmitt *et al* in 2016.^16^ The systematic review will be limited by searching for economic evaluation studies published in English; these factors might limit the generalisability of the findings. Another potential limitation of the systematic review might include the heterogeneity we expect to observe concerning study designs, extreme weather events, health conditions and population groups assessed, which might limit a powered subgroup analysis.
