# Supplementary material for: Protocol for a systematic review of economic evaluations considering costs and health outcomes of weather and climate-related extreme events in humans
Source: BMJ Open. 2025 Feb 7;15(2):e096554. doi: 10.1136/bmjopen-2024-096554 (PMC11808864; doi:10.1136/bmjopen-2024-096554)
Supplement: Supplementary file 1 [file bmjopen-2024-096554.reviewer_comments.pdf]

PEER REVIEW HISTORY

BMJ Open publishes all reviews undertaken for accepted manuscripts. Reviewers are asked to complete a checklist review form and are provided with free text boxes to elaborate on their assessment. These free text comments are reproduced below.

ARTICLE DETAILS

Title (Provisional)

Protocol for a systematic review of economic evaluations considering costs and health outcomes of weather and climate-related extreme events in humans

Authors

Babashahi, Saeideh; Iwuji, Collins; Orievulu, Kingsley; Eyita-Okon, Ekeminiabasi; Kniveton, Dominic

---

VERSION 1 - REVIEW

**Reviewer**	**1**
**Name**	**Mohsin, Muhammad**
**Affiliation**	**National University of Sciences and Technology, SINES**
**Date**	**13-Dec-2024**
**COI**	**None**

Dear Authors,

This work is very timely. I have few minor comments:

1. Climate events don't always impact health outcomes directly. Impact can be in other forms, for example: Due to change in weather patterns, crop of that year in a particular region did not grow as good as expected. Since reduced quantity/quality of crop is available, region goes through famine (maybe famine is too much) but let say population’s consumption of certain vitamins was reduced that year and that comes with a health economic cost.

of course above is a fictional situation, but would this review include such studies as well? If yes, you need to broaden the key terms (I understand given registered protocol, it won’t be straight forward)

2. About population: Not all populations are affected equally by Climate related events. For-example poor people in underdeveloped countries may suffer more as compared to rich from floods, not necessarily but this may happen. Would this review include perspective of different populations? (that maybe based on age, gender, socioeconomic status etc).

3. There is no restriction on geographic location, but review only includes literature in English language. Some literature from different regions may primarily in different languages (Chinese, Urdu, Russian etc) but translations possibly available in English. Would you be considering those translations?

I really appreciate the work being done, and looking forward to reading finding of review paper.

Regards,

**Reviewer**	**2**
**Name**	**Griffiths, Jenny**
**Affiliation**	**Health and Sustainability Network**
**Date**	**16-Dec-2024**
**COI**	**None**

This systematic review will meet an important need in the public health and health care communities for an up-to-date overview of what is known about the cost and health impacts of extreme weather events related to climate breakdown. The review of published literature itself, especially of grey literature, is likely to prove challenging, but that is a good reason to undertake it! The methodology proposed appears appropriate.

VERSION 1 - AUTHOR RESPONSE

Comments from Reviewer 1:

This work is very timely. I have few minor comments:

Authors' response: Thank you for your encouraging remarks and your comments,

Comment 1. Climate events don't always impact health outcomes directly. Impact can be in other forms, for example: Due to change in weather patterns, crop of that year in a particular region did not grow as good as expected. Since reduced quantity/quality of crop is available, region goes through famine (maybe famine is too much) but let say population's consumption of certain vitamins was reduced that year and that comes with a health economic cost.
of course above is a fictional situation, but would this review include such studies as well? If yes, you need to broaden the key terms (I understand given registered protocol, it won't be straight forward)

Authors' response: Thank you for your suggestion. We agree that climate-induced extreme weather events (EWEs) result in both direct and indirect health outcomes. As mentioned in the protocol, we will include any type of economic evaluation studies that assessed any (direct and indirect) economic and health-related outcomes of any type of EWEs.

Comment 2. About population: Not all populations are affected equally by Climate related events. For-example poor people in underdeveloped countries may suffer more as compared to rich from floods, not necessarily but this may happen. Would this review include perspective of different populations? (that maybe based on age, gender, socioeconomic status etc).

Authors' response: Yes, we agree that EWEs disproportionately affect certain population groups. As mentioned in the methods and Table 1, studies will be included with no restriction based on gender, age, socioeconomic status, health condition, context, geographic area, etc.

Comment 3. There is no restriction on geographic location, but review only includes literature in English language. Some literature from different regions may primarily in different languages (Chinese, Urdu, Russian etc) but translations possibly available in English. Would you be considering those translations?

Authors' response: Thank you for your suggestion. We will include studies without restriction on geographical location subject to the availability of the study in English. Unfortunately, we don't have the resources to include studies published in other languages and this has been included as one of the limitations of the systematic review.

I really appreciate the work being done, and looking forward to reading finding of review paper.

Authors' response: Thank you so much.

Comments from Reviewer 2:

This systematic review will meet an important need in the public health and health care communities for an up-to-date overview of what is known about the cost and health impacts of extreme weather events related to climate breakdown. The review of published literature itself, especially of grey literature, is likely to prove challenging, but that is a good reason to undertake it! The methodology proposed appears appropriate.

Authors' response: Thank you for your encouraging remarks.
